# Effect and comparative analysis of urease inhibitor AHA and low pH method on MICP reinforcement of calcareous sand

**DOI:** 10.3389/fmicb.2025.1524693

**Published:** 2025-03-27

**Authors:** Jun Hu, Yahui Zhan, Yufei Yang, Zetian Li, Zhaokui Sun, Yongchang Yang, Shuai Zhang

**Affiliations:** ^1^School of Civil Engineering and Architecture, Hainan University, Haikou, China; ^2^Collaborative Innovation Center of Marine Science and Technology, Hainan University, Haikou, China; ^3^Hainan Institute of Hydrological and Geological Engineering Exploration, Haikou, China; ^4^School of Information and Communication Engineering, Hainan University, Haikou, China

**Keywords:** AHA method, MICP, low pH method, comparative analysis, mechanism of action

## Abstract

In order to alleviate the problem of grouting blockage caused by the immediate action of bacteria and chemical reagents in the process of MICP reinforcement of sandy soil, this paper delays the generation rate of calcium carbonate by adding urease inhibitor AHA to the cementing solution and lowering the pH of the bacterial solution. The mechanism of action of the two methods was explored, and their effects were compared and analyzed. The results indicate that both AHA method and low pH method can effectively delay the generation rate of calcium carbonate and improve the reinforcement effect of MICP under different mechanisms of action. The reinforcement effect is best when the AHA content is 0.1% or when the pH drops to 5.5. Comparing the two methods, it was found that the solidification effect of low pH method on sandy soil was slightly higher than that of AHA method. This is because the calcium carbonate crystal size generated by low pH method is smaller than that of AHA method. Under similar calcium carbonate content, the smaller the crystal size, the more bonding points between calcium carbonate and soil, and the higher its strength. Exploring the mechanisms of action of the two methods, it was found that the AHA method delays the rate of urea hydrolysis catalyzed by urease. The low pH method acts on the binding process of carbonate ions and calcium ions after urea hydrolysis, as well as inhibiting urease activity. The two methods have different mechanisms of action, but both can achieve the goal of delaying the rate of calcium carbonate formation.

## 1 Introduction

At present, the application of microorganism to solve environmental pollution and engineering problems has become a hot spot. For example, the micro-induced calcium carbonate deposition (MICP) technology can be used for the repair of heavy metals to protect the environment and human health (Xue et al., 2024a,b). In addition, it can also be used for soil solidification due to its high efficiency, low carbon and environmental protection, so as to meet the construction requirements of the project (Zhang et al., 2023; Fouladi et al., 2023). The reinforcement methods of MICP include urea hydrolysis (Achal and Pan, 2011), denitrification (Pham et al., 2016), sulfate reduction (Braissant et al., 2007), iron reductio (Weaver et al., 2012), etc. The reinforcement mechanism of MICP based on urea hydrolysis is clear, and it can quickly react to generate a large amount of calcium carbonate crystals, which is a research hotspot in microbial geotechnical studies (Gebru et al., 2021; Siddique and Chahal, 2011). The reinforcement principle can be divided into three stages: (1) Microbial metabolism secretes intracellular urease, which hydrolyzes urea to produce NH_4_^+^ and CO_3_^2−^. (2) CO_3_^2−^ combines with Ca^2+^ in the environment to form CaCO_3_ precipitate. (3) CaCO_3_ precipitation fills the particle pores and binds the loose soil (De Muynck et al., 2010; Peng et al., 2020). MICP reinforcement involves complex biochemical reactions, and there are many factors that affect its reinforcement effect. Wang et al. (2023)conducted urease activity tests at different temperatures and found that low temperature (4°C) did not reduce urease activity, but limited the final amount of cementation. Under high temperature conditions (50°C), urease activity will significantly decrease in a short period of time. At this time, adopting a grouting scheme of one bacterial solution and two cementing solutions can increase the final bacterial cementing amount. Wen et al. (2020) found that the higher the initial concentrations of bacteria and urease, the faster the reaction rate of CaCO_3_ precipitation. Al Qabany and Soga (2014) studied the reinforcement effect of mixed solutions of urea and calcium chloride at different concentrations, and the results showed that lower concentration solutions could obtain smaller and more uniform calcium carbonate crystals covering the contact area of sand particles. Cheng et al. (2013) found that MICP cured fine sand samples have higher cohesion and smaller internal friction angles than coarse sand samples, and smaller soil particles have more calcium carbonate crystal contact points between them, resulting in better curing effects.

Previous studies have found that temperature, properties of bacterial solution, concentration of cementing solution, and particle size of sand can all affect the reinforcement effect of MICP. But fundamentally, it is mainly the amount of calcium carbonate generated and the distribution of calcium carbonate in sandy soil that ultimately determine the strength of the soil. However, the traditional MICP reinforcement method, when injecting bacterial and cementing fluids into the sand column, produces a large amount of calcium carbonate flocs that quickly block the pores near the injection end. The cementing fluid is difficult to evenly distribute in the sand column, resulting in uneven distribution of calcium carbonate generated at the lower end of the sand column, and insufficient strength at the lower end of the sample, making it more susceptible to damage. Therefore, in order to slow down the rate of calcium carbonate formation, Whiffin et al. (2007) developed a biphasic injection method, which involves injecting bacterial solution first and then cementing solution. The advantage of this injection strategy is that it avoids rapid flocculation and blockage of pores near the injection end; Cheng et al. (2019) found that reducing the pH value of the bacterial solution in advance can cause a lag period in the biological bonding process and prevent blockage caused by biological flocculation. Xu et al. (2020) found that injecting a certain amount of NBPT urease inhibitor into the cementing solution to reinforce quartz sand can delay the time of urea hydrolysis and biological flocculation.

It is worth noting that the previous application of urease inhibitor in MICP solidified sand is less and mostly limited to a single type, and no systematic study has been formed. Therefore, in this study, acetohydroxamic acid (AHA) was selected innovatively as a urease inhibitor. As an effective urease competitive inhibitor, AHA can react with nickel ions in the urease activity center, so as to inhibit the urease activity and effectively delay the urea hydrolysis process. In addition, the research object of solidified sand is calcareous sand. Previous studies mostly focus on conventional terrigenous sand. However, calcareous sand is fragile and has low strength, which is significantly different from terrigenous sand. Therefore, research on calcareous sand is more challenging and has practical application value. In this paper, the urease inhibitor AHA method and low pH method are, respectively, applied to the reinforcement of calcareous sand, and the physical properties, mechanical properties and microscopic tests of the reinforced calcareous sand are compared and analyzed to reveal the different reinforcement mechanisms of the two methods.

## 2 Materials and methods

### 2.1 Experimental materials

**Figure 1:**
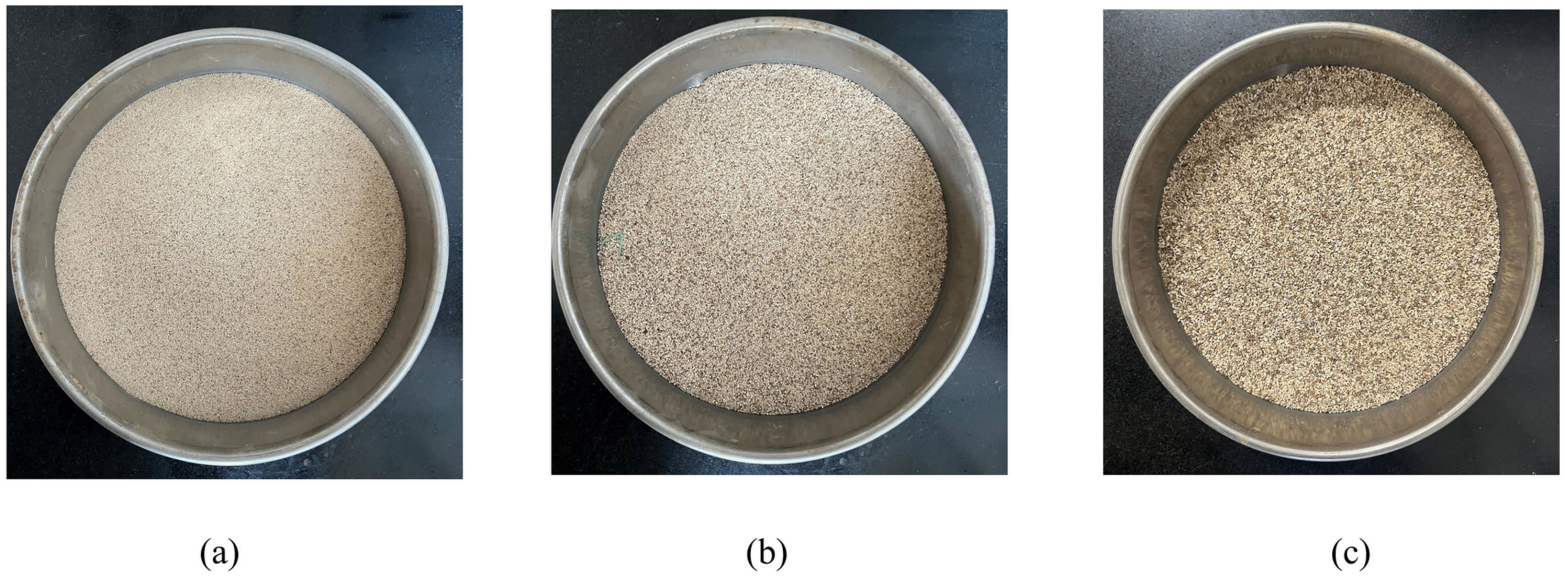
Experimental sand particles; **(a)** 0.5 mm; **(b)** 0.5–1.0 mm; **(c)** 1.0–2.0 mm.

**Figure 2:**
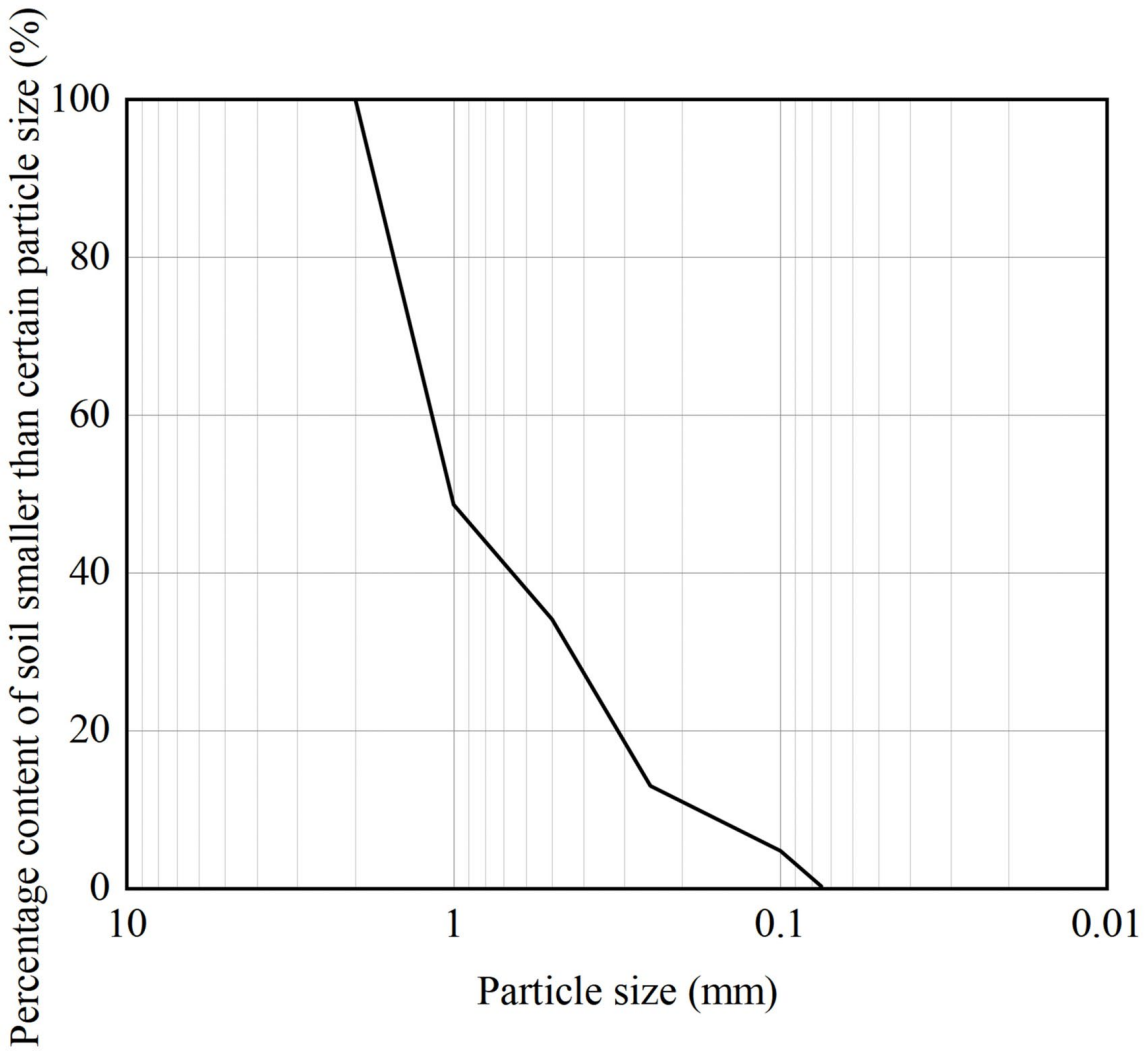
Grain size distribution curve.

The sand used in this experiment was taken from an island in Sansha City, Hainan Province. After cleaning, drying, and cooling the original sand sample, some impurities were removed to obtain the test sand as shown in Figure 1. The physical parameter indicators of the calcareous sand are shown in Table 1. The parameter index determination process is carried out in accordance with the Chinese soil test method standard (GB/T50123-2019), which is applicable to this test and the calculation results are reliable. The sand particle size range used in this experiment is below 2 mm. Figure 2 shows the grain composition curve of calcareous sand used in this test. The grain size of calcareous sand is less than 2 mm, and the sand with particle size range has important application value in coastal engineering and soil improvement (Li et al., 2025). Therefore, the calcareous sand with particle size less than 2 mm selected in this paper is closer to the actual engineering. After grinding the experimental sand into powder, X-ray diffraction (XRD) and X-ray fluorescence spectroscopy (XRF) were conducted to determine the specific composition of the sand. The main component is calcium carbonate, as shown in Table 2.

**Table 1:** Basic physical performance indicators of experimental sand.

Parameter	Non-uniformity coefficient	Curvature coefficient	Particle specific gravity	Maximum dry density (g/cm^3^)	Minimum dry density (g/ cm^3^)	Maximum void ratio	Minimum porosity ratio
Value	4.484	0.76	2.618	1.702	1.379	0.91	0.54

**Table 2:** Specific composition of experimental sand.

Mineral type	CaCO_3_	SiO_2_	Ca_2_(SO_4_)_2_(H_2_O)	Other
Mineral content	62.9%	24.5%	7.6%	5%

**Figure 3:**
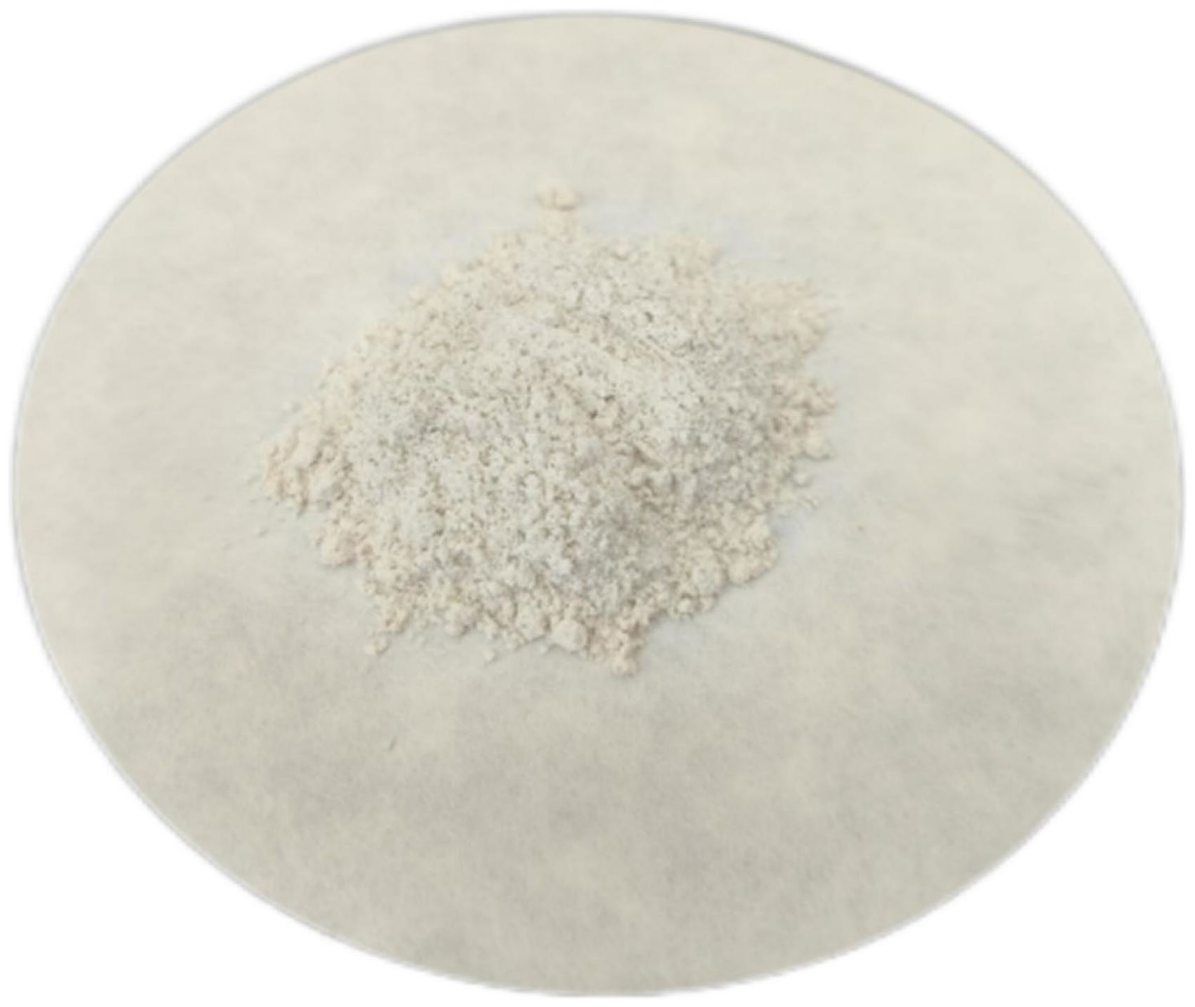
Acetohydroxamic acid.

Urease inhibitors can regulate the activity of urease. The urease inhibitor selected in this experiment is acetohydroxamic acid (AHA) (Siddique and Chahal, 2011), as shown in Figure 3. Among the types of urease inhibitors, it belongs to competitive inhibitors. Competitive inhibitors mainly act on the nickel ions in the active center of urease, causing urea to be unable to bind with urease normally and undergo hydrolysis (Follmer, 2010; Amtul et al., 2002).

### 2.2 Preparation of bacterial solution and cementing solution

The experiment selected *Bacillus subtilis* as the strain for MICP reinforcement. *Bacillus subtilis* produces intracellular urease through metabolism, providing conditions for urea hydrolysis. Place *Bacillus subtilis* in a conical flask containing yeast extract liquid medium (bacteria: medium = 1:50), and incubate the flask in a constant temperature oscillation box (temperature 30°C, speed 190 r/min) for 24 h. Bacterial concentration is characterized by measuring the optical density (OD600) of bacterial solution at a wavelength of 600 nm using a spectrophotometer. By using a conductivity meter to detect the conductivity of ions in the solution, urease activity can be characterized, indirectly reflecting bacterial activity (Whiffin, 2008). In this test, take 2 mL bacterial solution and mix it with 18 mL urea solution in a small beaker. Use a conductivity tester to record the average change in conductivity per minute, and multiply the dilution multiple to obtain the urease activity, in mS/cm/min. The calculation formula of urease activity is:


Urease activity=ΔEC×Dilution factor

ΔEC: average conductivity variation per minute.

The average urease activity used in this experiment was 1.43 mS/cm/min, and the average OD600 value measured was 1.61.

The cementing solution used in the experiment was prepared by mixing urea and calcium chloride in a 1:1 volume ratio (Gorospe et al., 2013; Al-Salloum et al., 2017), with the concentrations of urea and calcium chloride set at 1 mol/L. Urea plays a role in providing carbon and energy sources for bacteria during this process, while calcium chloride provides a calcium source, allowing the carbonate ions produced by urea hydrolysis to combine with calcium ions to form calcium carbonate precipitates and bond between sand particles.

### 2.3 Preparation and reinforcement of samples

**Figure 4:**
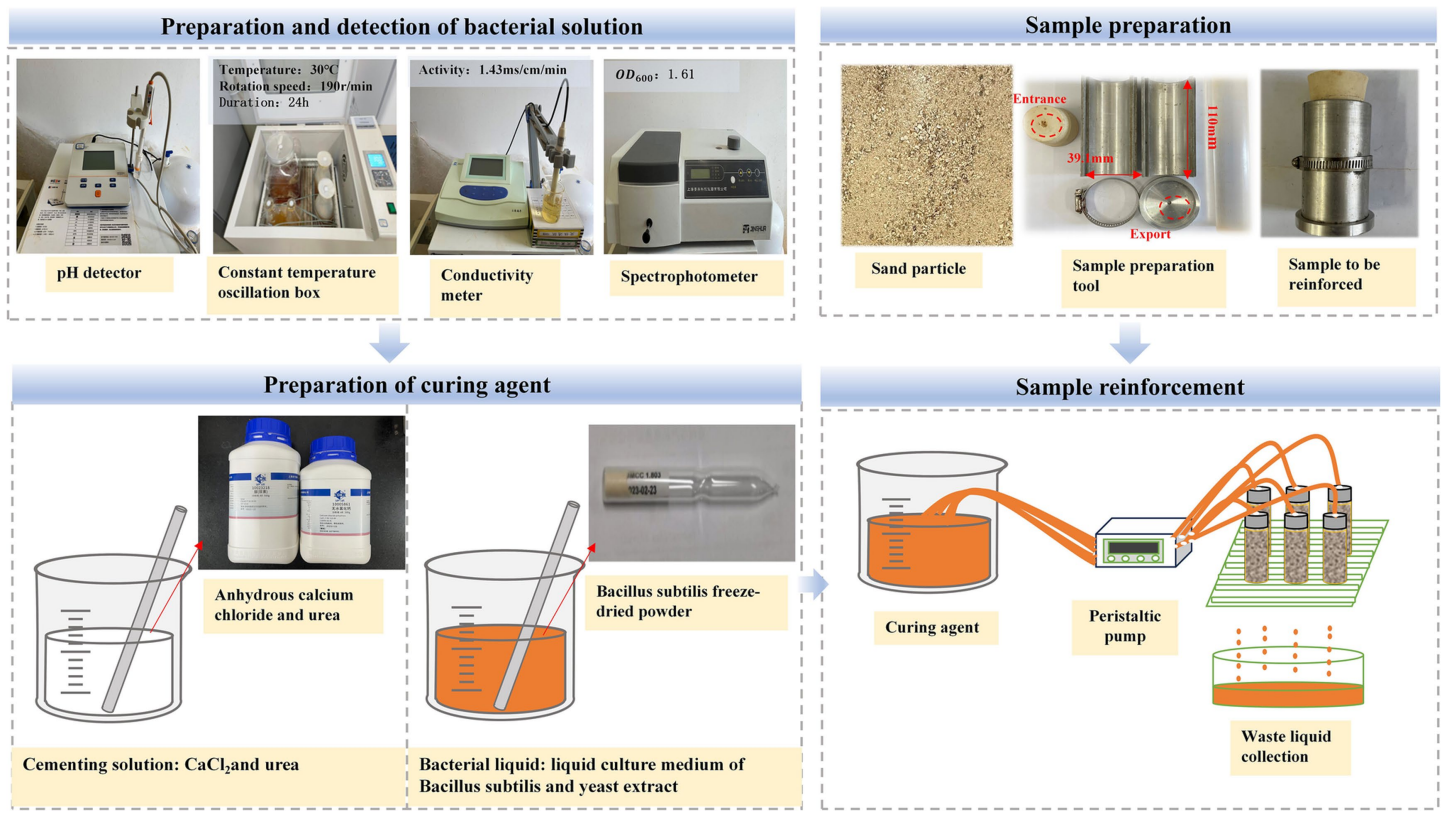
Sand samples reinforcement process.

According to the Standard of Geotechnical Test Methods (GB/T50123-2019), stainless steel molds with a sample diameter of 39.1 mm and a height of 110 mm were customized. Samples with a size of 39.1 mm × 80 mm were prepared. The mold is a double-open, and the embedding part of the mold adopts zigzag splicing and is closely connected with the perforated base to ensure that the liquid does not leak at the joint during the microbial reinforcement process. Firstly, the influence of AHA on the reinforcement effect of MICP was explored. Different contents of AHA were added to the cementation solution, with the dosage expressed as a percentage of the mass of urea, namely 0, 0.05, 0.1, 0.25, 0.5, and 1% (Xu et al., 2020). Five parallel samples are set in each group. The sample was injected with 50 mL (about 1 pore volume) of bacterial liquid from the upper end at a speed of 5 mL/min by peristaltic pump and then stood for 3 h. After 3 h, 50 mL cementing fluid (with different contents of AHA) was injected into the sand column at the same speed. After standing for 12 h, the bacteria solution and cementing solution are repeated for one round of grouting, and the grouting period lasts for 8 days. The sample was removed from the mold after 8 days and immersed in deionized water for 24 h to remove impurities and soluble salts from the sample surface and reduce experimental errors. Finally dried in a 55°C oven for 48 h. The low pH method is to adjust the pH value of bacterial solution to 6.5, 6, 5.5, 5 and 4.5 with hydrochloric acid. At the same time, for comparative analysis, a group of conventional reinforcement tests using the original pH value (pH = 8.5) were set up. Other conditions remain unchanged, and the reinforcement method is the same as that of AHA method. Figure 4 is a sample reinforcement flow chart. First, the preparation and detection of the bacterial solution, the concentration and activity of the prepared bacterial solution are detected and recorded (the preparation method is completed according to the requirements of 2.2); Secondly, the calcium chloride and urea are mixed in proportion to make the cementing liquid for standby. In addition, prepare the calcareous sand required for the test into the mold, and connect the configured bacterial fluid and cementing fluid with the peristaltic pump to inject the sample according to the requirements described in 2.3 to complete the reinforcement.

### 2.4 Testing program

In order to verify the effectiveness of AHA method and low pH method in sand MICP reinforcement, a series of physical and mechanical property tests were conducted on the reinforced sand samples, including calcium carbonate generation rate, water absorption rate, unconfined compressive strength (UCS), as well as observation of failure mode and microstructure analysis. Through these experiments, the effectiveness of two methods in reinforcing sand was compared and analyzed, providing experimental evidence for practical engineering.

## 3 Results and discussion

### 3.1 Calcium carbonate formation rate

**Figure 5:**
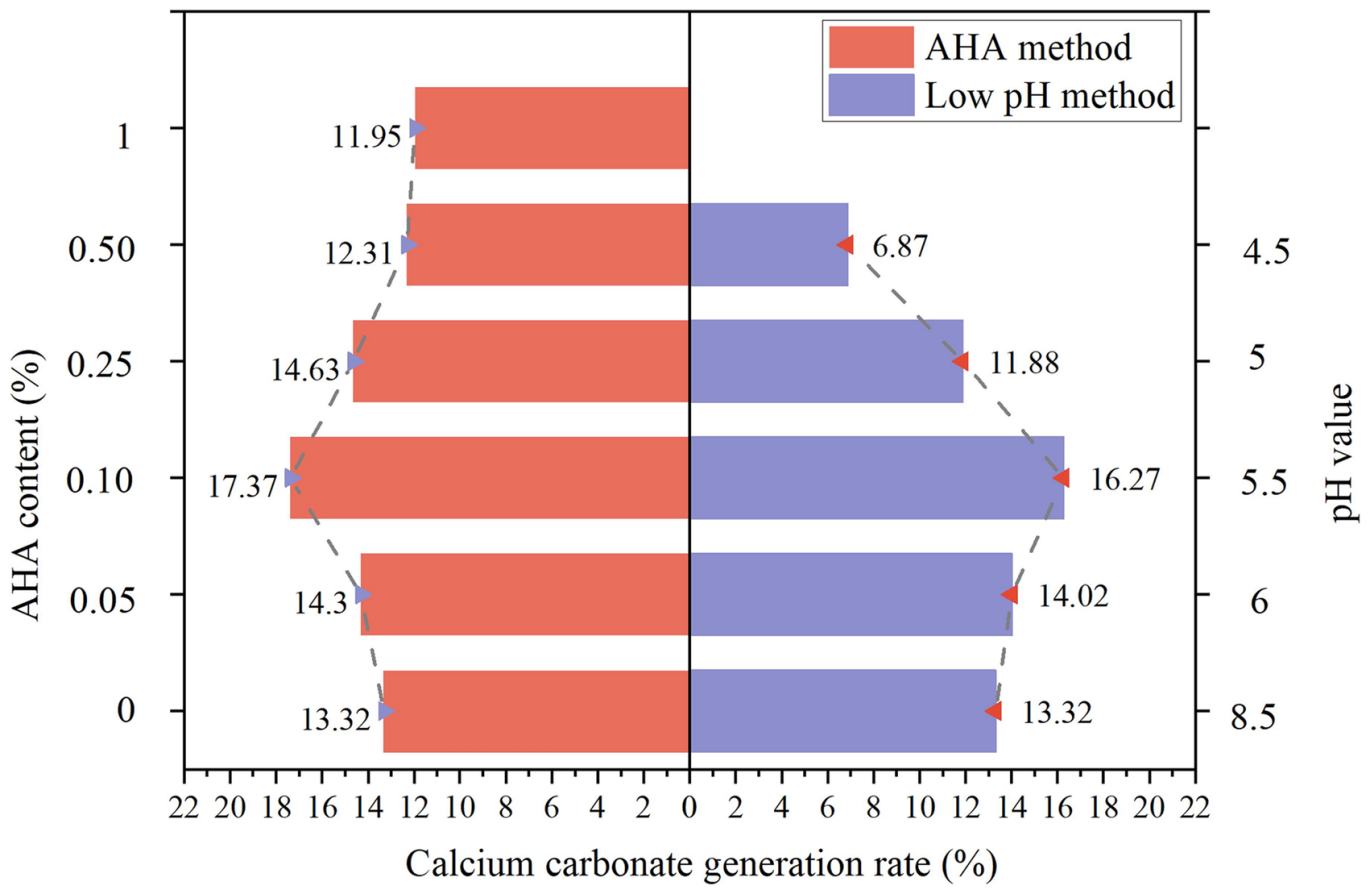
Production rate of calcium carbonate by AHA method and low pH method.

Figure 5 illustrates the variation trend of calcium carbonate formation rate in samples consolidated by the AHA method and the low pH method. From the left side of Figure 5, it can be observed that with the increase of AHA content, the formation rate of calcium carbonate in the sample shows a trend of increasing at first and then decreasing. This is because a small amount of AHA is added at the beginning to have a certain inhibitory effect on part of the urease in the bacterial solution, while the other part of the urease can still react with urea. At this time, it can relieve the accumulation phenomenon caused by quick generation of calcium carbonate at the upper end of the sample, so that the bacterial solution and the cementing liquid are evenly distributed in the sample, and the bacterial solution and the cementing liquid can be fully reacted. Therefore, with the increase of AHA content, the more sufficient the reaction is, the more calcium carbonate content is. However, when the AHA content reaches the critical value, the generation of calcium carbonate reaches the maximum. Too much AHA content will excessively inhibit the urease activity, making urea difficult to be hydrolyzed under the action of urease. Therefore, when the AHA content exceeds the critical value, the calcium carbonate content will decline. When AHA was not added, the calcium carbonate production rate of the sample was 13.32%, at this time, a large amount of calcium carbonate was accumulated at the upper end of the sample, and the calcium carbonate content at the lower end of the sample was less. When the AHA content is 0.05–0.25%, it has a certain promotion effect on the calcium carbonate formation rate, especially when the AHA content is 0.1%, the calcium carbonate production rate reaches the highest, which is 17.37%; However, when the content of AHA is further increased to 0.5–1%, its influence on the calcium carbonate production rate in the sample changes to inhibition. At this time, when the content of AHA is too high, most urease activities are inhibited. At this time, the calcium carbonate production rate is lower than that without AHA, so the experimental data show that adding an appropriate amount of AHA can promote the production of calcium carbonate.

**Figure 6:**
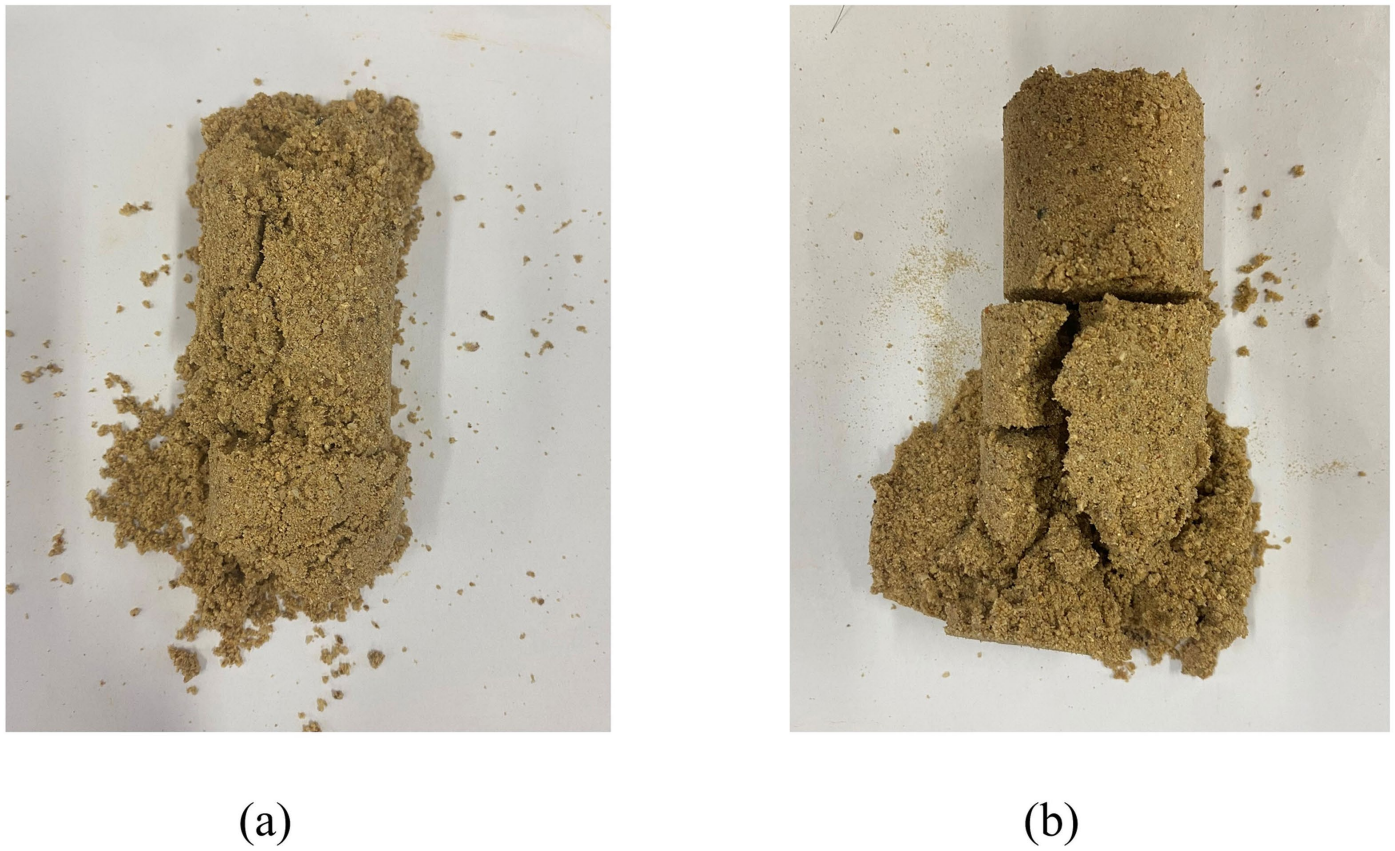
Unformed calcareous sand. **(a)** pH = 4; **(b)** pH =6.5.

From the right side of Figure 5, it can be observed that the calcium carbonate production rate after the sand sample is reinforced by bacterial solution with pH value of 4.5–6 (The reinforcement of the sample was unsuccessful when pH = 4 and pH = 6.5), and the calcium carbonate production rate in the sand column shows a trend of increasing at first and then decreasing. Figure 6 presents the schematic diagram of the unconsolidated sample. When the pH value of bacterial liquid drops to 6.5, the sand column appears the phenomenon that the reinforcement effect is successful at the upper end and unsuccessful at the lower end. This shows that when the pH value of the bacterial liquid is reduced to a small extent, the reaction speed of the bacterial liquid and the cementing liquid in the sample cannot be effectively delayed, resulting in uneven reinforcement effect and failure to form a sample. At the same time, when the pH value drops to 4, the sand column cannot form added solids, because the pH value of the bacterial solution is too low, and carbonate ions cannot exist in a large number in the acidic solution, so there is basically no precipitation of calcium carbonate in the sand column. When the pH value of bacterial liquid is 5.5, the highest calcium carbonate production rate can be obtained, which is 1.23 times of the original pH value. Further observation shows that when the pH value is adjusted to 6, the calcium carbonate production rate is lower than that when the pH value is 5.5, but it is still higher than that of the original pH group.

### 3.2 Water absorption rate

**Figure 7:**
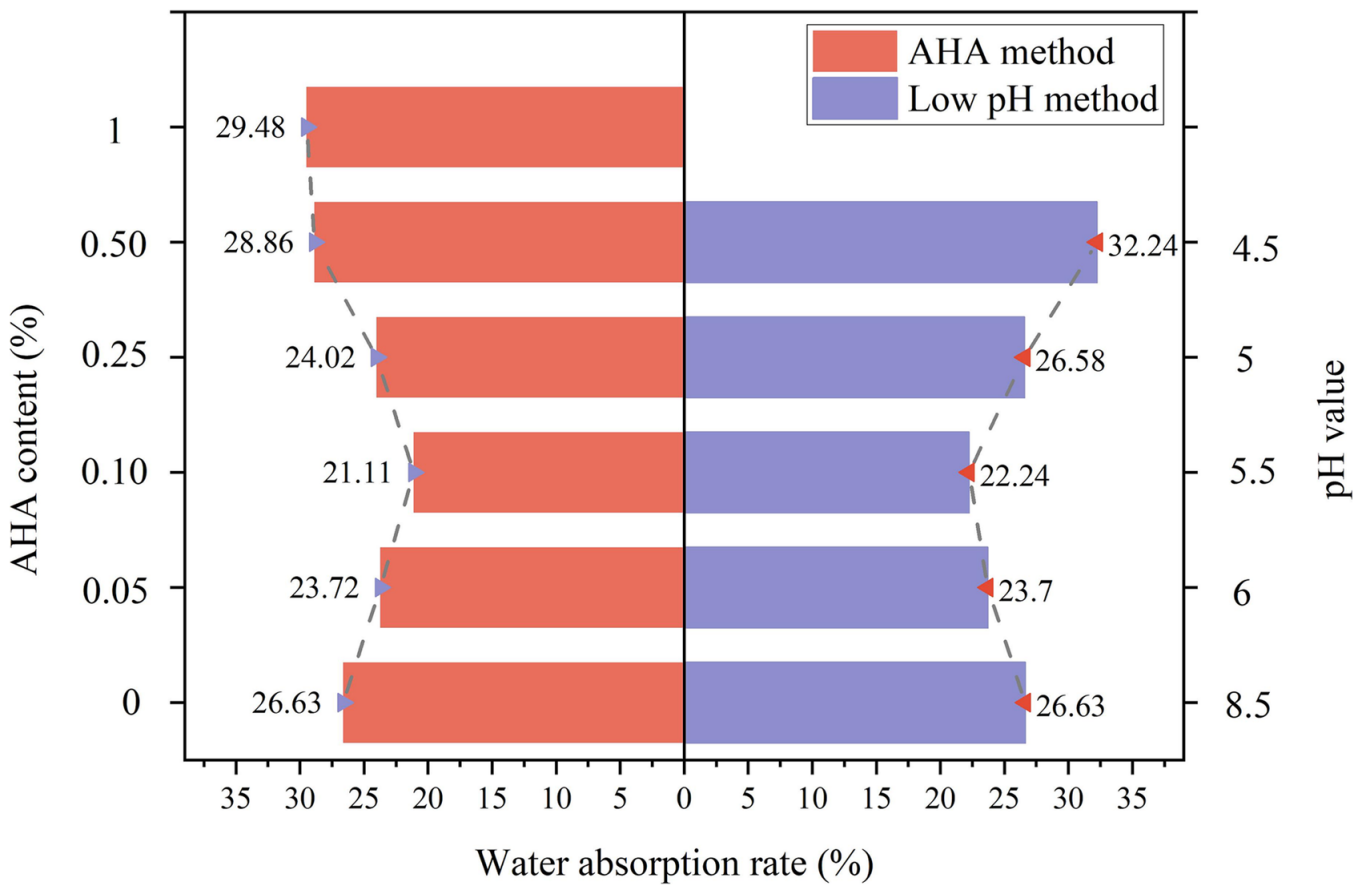
Water absorption rate of AHA method and low pH method.

Figure 7 depicts the variation trend of water absorption rate in samples consolidated by the AHA method and the low pH method. The development trend of water absorption rate and calcium carbonate generation rate is opposite. This is because the generated calcium carbonate can fill the pores between calcareous sand particles. The more calcium carbonate is generated, the smaller the porosity of the sand column, and the lower the water absorption rate. On the contrary, the less calcium carbonate is generated, which is not enough to fully fill the pores between sand particles. At this time, the porosity of the sand column is higher, and the water absorption rate is also higher. When the AHA content is 0.1%, the water absorption rate reaches the lowest value of 21.11%. When the pH value of the bacterial solution decreases to 5.5, the lowest water absorption rate is 22.24%.

### 3.3 Unconfined compressive strength

#### 3.3.1 Peak of unconfined compressive strength

**Figure 8:**
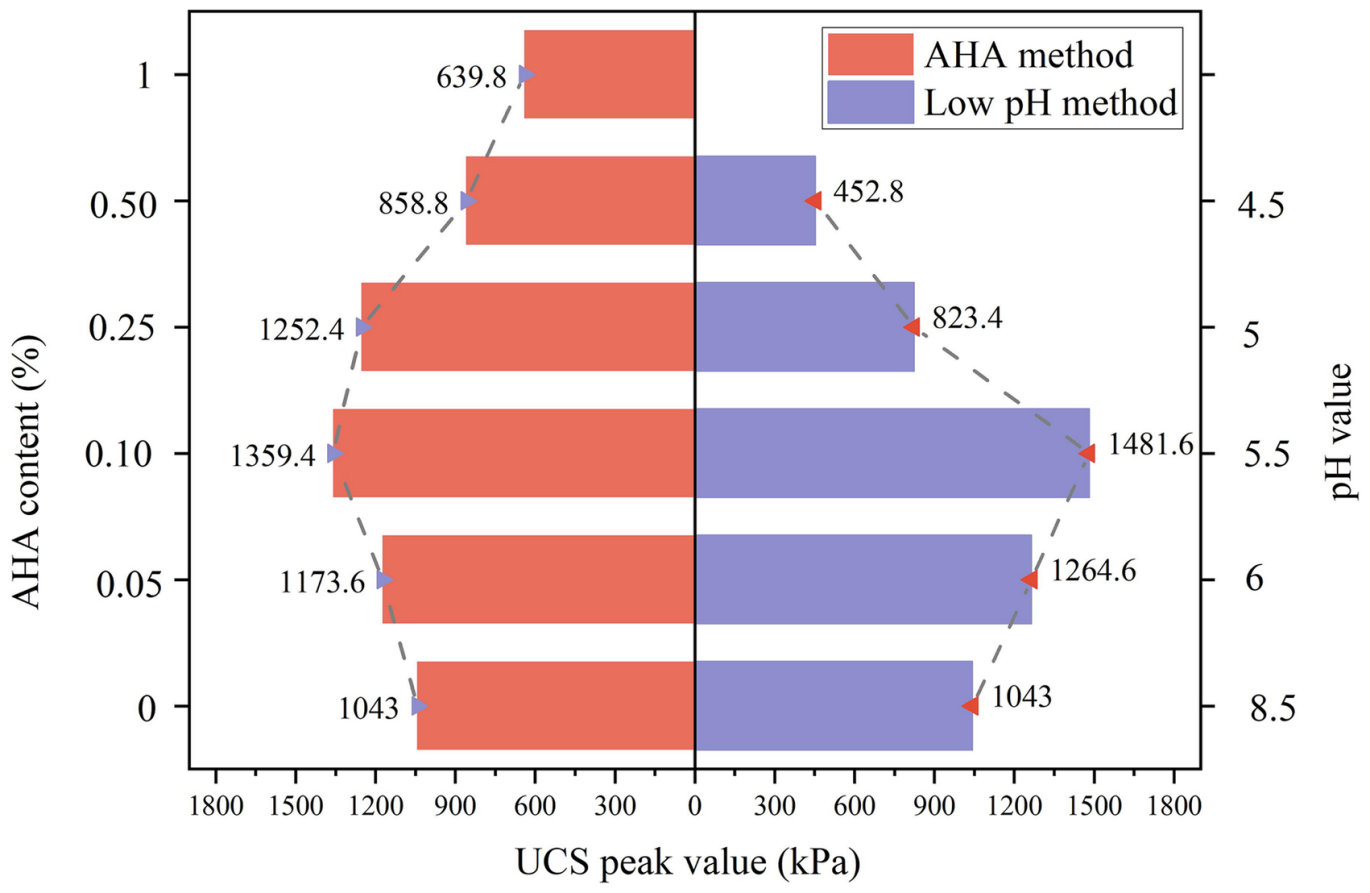
Peak value of UCS of AHA method and low pH method.

Figure 8 shows the unconfined compressive strength after curing by AHA method and low pH method. As shown on the left side of Figure 8, when AHA is not added, the peak of UCS value of the sample is 1,043 kPa. At this time, the upper end of the sample is blocked due to the accumulation of calcium carbonate, resulting in less generation and uneven distribution of calcium carbonate at the lower end of the sample, which is damaged; When adding AHA content of 0.05–0.1%, the generation rate of calcium carbonate is effectively delayed, avoiding blockage of the injection port and allowing the bacterial and cementing fluids to penetrate more evenly into the sand sample. Therefore, the amount of calcium carbonate generated at the lower end of the sample increases, which better bonds the sand particles and improves the UCS peak value. When the AHA content is 0.1%, the peak of UCS reaches 1359.4 kPa. And it rapidly decreases within the range of 0.25–0.5%, even lower than the unconfined compressive strength at zero addition. When AHA was added and increased to 1%, the peak strength of the sand sample was 0.61 times that without AHA, and only 0.47 times that with 0.1% AHA added. Therefore, if the AHA content is too high, the overall calcium carbonate generation rate of the sample is low, and the strength of the sample is not high, making it easy to be compressed and damaged.

As shown on the right side of Figure 8, adjusting the pH value of the bacterial solution to the range of 5.5–6 can significantly enhance the UCS peak of the sample. At a pH value of 5.5, the reinforcement effect is optimal, with an unconfined compressive strength of 1481.6 kPa, which is 1.43 times higher than the peak strength of the original pH value (control group). However, when the pH value of the bacterial solution drops to the range of 4.5–5.5, the reinforcement effect is significantly lower than the original pH value. Especially in the sample with a pH value of 4.5, the peak intensity is only 452.8 kPa, which is only 0.34 times the peak intensity of the original pH sample. These results indicate that excessively reducing the pH value of the bacterial solution may greatly inhibit the reaction between the bacterial solution and cementing solution and the sand, thereby negatively affecting the reinforcement effect.

**Figure 9:**
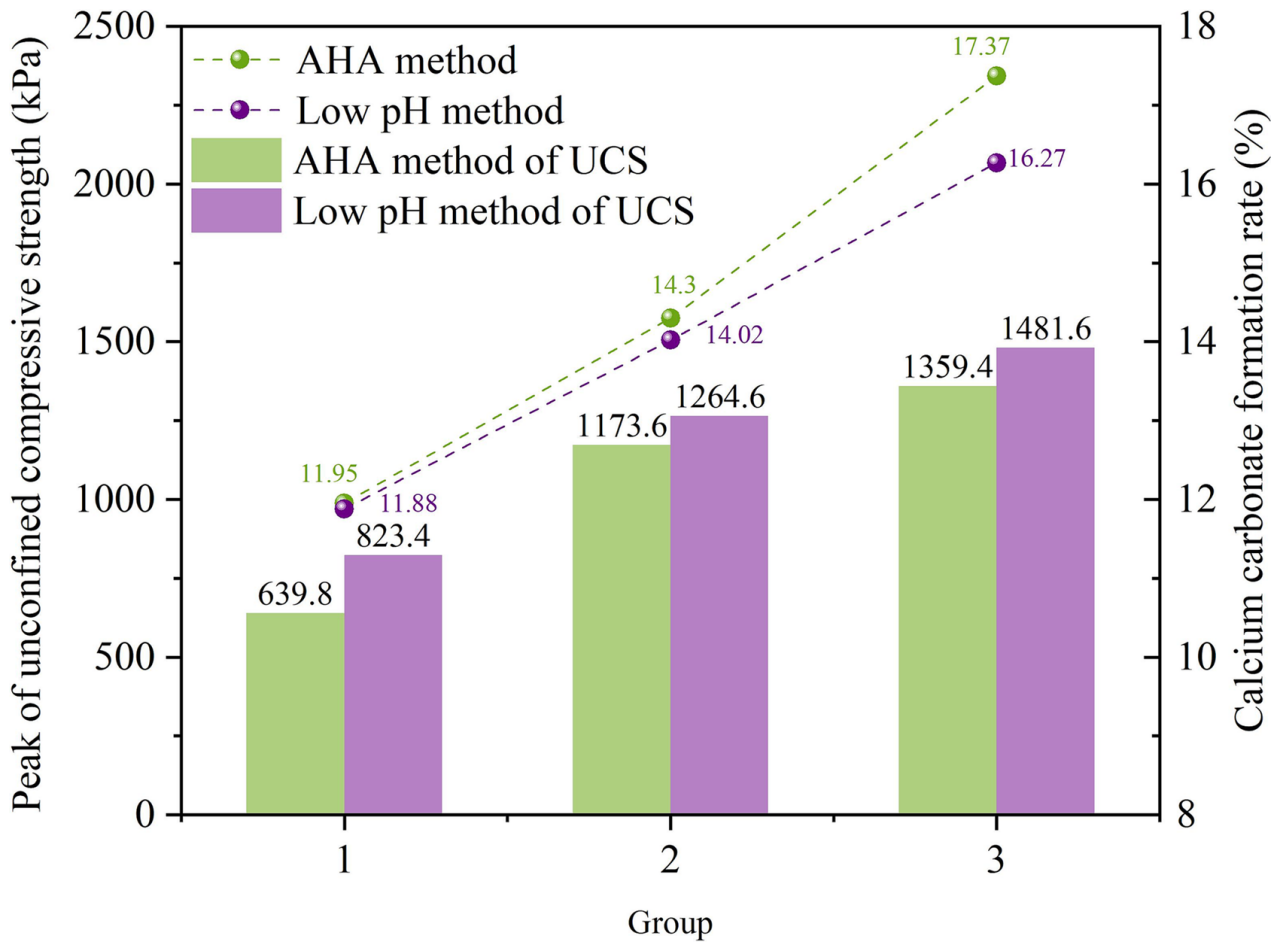
Comparison of UCS between AHA method and low pH method.

In order to compare the reinforcement effects of AHA method and low pH method, this study selected three groups of sand columns with similar calcium carbonate generation rates for comparative analysis of unconfined compressive strength, as shown in Figure 9. Specifically, the selection of the three groups of samples is as follows: Samples containing 1% AHA and pH = 5 were used as the first group. Samples with 0.05% AHA and pH = 6 were selected as the second group. The third group consists of samples with 0.1% AHA and pH = 5.5. Taking Group 2 as an example, the content represented in the bar chart is the rate of calcium carbonate formation. From the graph, it can be observed that the calcium carbonate generation rate obtained by the AHA method is slightly higher than that of the low pH method, but its unconfined compressive strength is lower than the latter. This is because, under similar amounts of calcium carbonate formation, the low pH method generates calcium carbonate crystals that are smaller in size and more numerous compared to the AHA method. The increased number of bonding points between calcium carbonate and sand particles leads to higher strength. Specifically, under the optimal conditions of AHA content and pH value, as shown in the third set of data in the figure, the UCS peak of the AHA method is 91.75% of that of the low pH method.

This result indicates that under the same conditions of calcium carbonate generation rate, the reinforcement effect of AHA method is slightly lower than that of low pH method. However, it still played a significant promoting role in reinforcing calcareous sand, and compared to the low pH method, it had a smaller impact on the soil environment.

#### 3.3.2 Stress–strain curves

**Figure 10:**
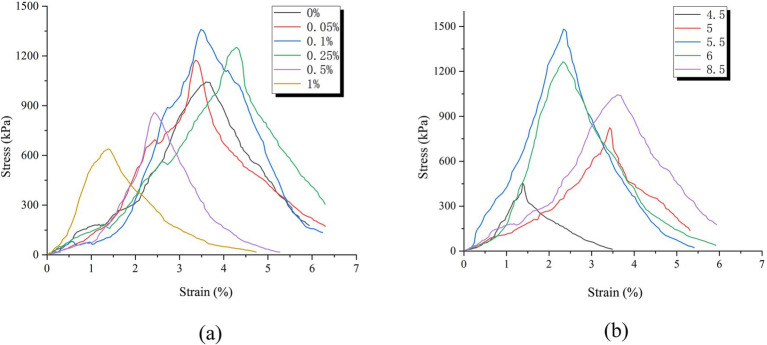
Stress–strain curve of different reinforcement methods. **(a)** Stress–strain curve of AHA method. **(b)** Stress–strain curve of Low pH method.

As shown in Figure 10, the specimens reinforced by the two methods exhibit similar characteristics on the stress–strain curve of unconfined compressive strength. In the initial stage of vertical compression, stress and strain show a positive correlation trend, and strain increases rapidly. When the peak stress is reached, the strain rapidly decreases and the specimen undergoes brittle failure along the main failure surface. Overall, it can be divided into three stages: during the elastic stage, the internal pores of the specimen are gradually compressed in the initial stage of compression, becoming denser, allowing loose calcareous sand particles to bond into a hard skeleton through the structure of “calcareous sand—calcium carbonate—calcareous sand.” Therefore, even under vertical pressure, the specimen will not immediately fail, forming a trend of linear positive increase in stress and strain. During the damage stage, as the vertical pressure increases, microcracks first appear in the weak areas where there is less calcium carbonate crystallization inside the sample. Subsequently, the continuous increase in internal stress in the sample destroys the hard skeleton of “calcareous sand—calcium carbonate—calcareous sand,” causing local damage to the sample, resulting in peak stress; In the final stage of failure, further compression after the peak stress occurs causes a large number of cracks to form inside the specimen, which penetrate each other and form the main failure surface. At this point, the bonding force inside the sample completely fails, and the stress rapidly decreases, resulting in a significant decrease in overall strength.

### 3.4 Damage mode analysis

**Figure 11:**
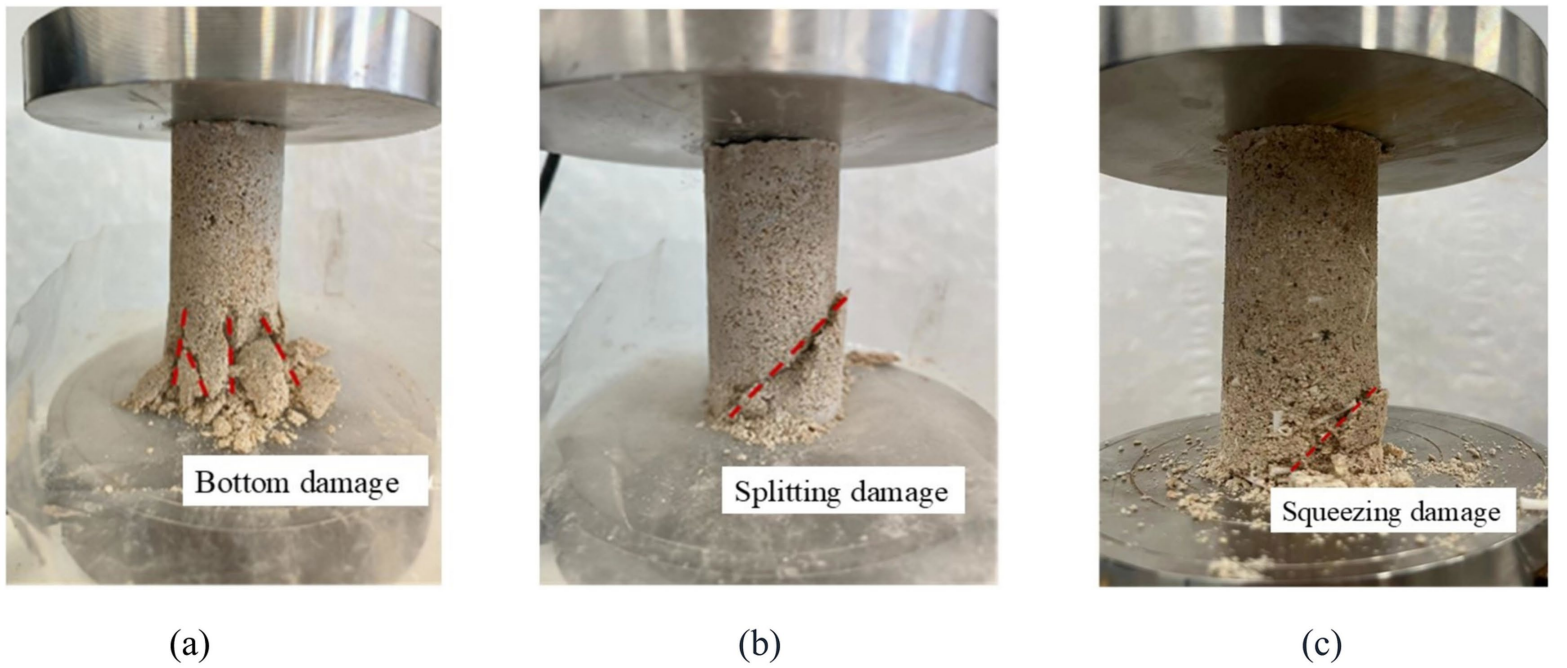
Damage modes of different reinforcement methods. **(a)** Control group. **(b)** AHA method (content is 0.1%). **(c)** Low pH method (pH = 5.5).

Figure 11 shows the schematic diagram of failure modes for different consolidation methods. From Figure 11, it can be seen that the failure mode of the reinforced samples in the control group (without adding AHA and changing pH value), with the addition of AHA and low pH methods, mainly manifested as lower part failure. The reason for this phenomenon is that all three methods use a top-down injection method, which allows the bacterial and cementing fluids to first come into full contact with the upper end of the sample and generate calcium carbonate crystals here. These calcium carbonate crystals filled the internal pores at the upper end of the sample, hindering the flow of bacterial and cementing fluids to the lower part, resulting in uneven distribution of calcium carbonate in the sample. Therefore, the upper end of the specimen has a stronger ability to withstand vertical loads compared to the lower end, while the lower end becomes the weak surface of the bond. In specimens reinforced by conventional methods, multiple cracks will appear at the lower end and form multiple failure surfaces; In the specimens reinforced with AHA, the main damage was lower splitting, and even after cracking, they could be basically spliced into a complete structure; The samples reinforced by low pH method have fewer failure surfaces, mainly due to local damage in the lower part.

### 3.5 Microscopic comparison

**Figure 12:**
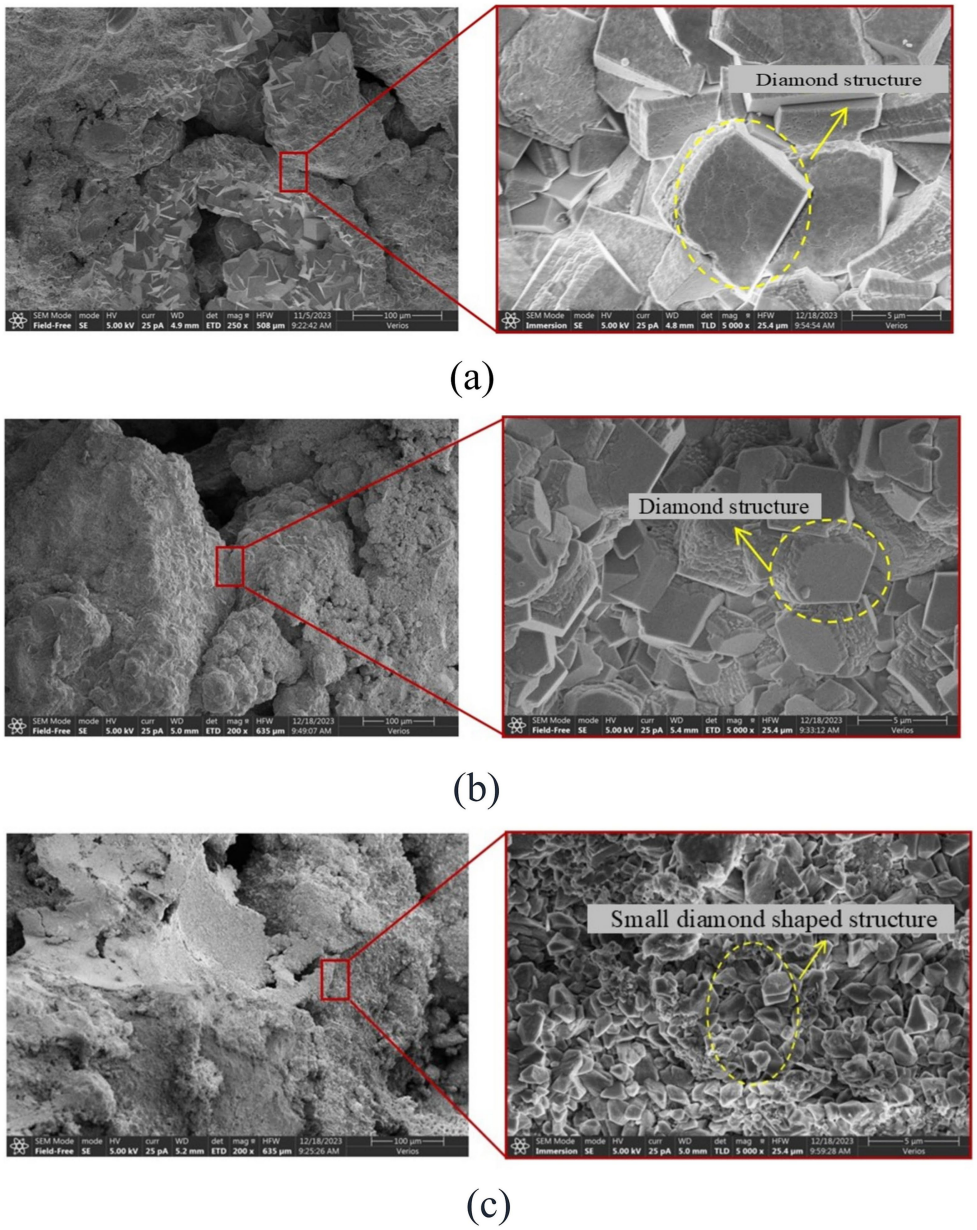
SEM scanning images of different reinforcement methods. **(a)** Control group. **(b)** AHA method (content is 0.1%). **(c)** Low pH method (pH = 5.5).

Samples with similar calcium carbonate content and different reinforcement methods were used for SEM testing, and were photographed at the same magnification of 5,000X, as shown in Figure 12. During the MICP reinforcement process, regardless of the control group, AHA method, or low pH method, the shape of the calcium carbonate crystals formed remained diamond shaped. However, in terms of crystal size, the calcium carbonate crystals reinforced by low pH method had the smallest size, followed by the method of adding AHA, while the calcium carbonate crystals reinforced by the control group had the largest size. The reason for this phenomenon is that there is a competitive relationship between the nucleation and growth of calcium carbonate crystals during the MICP process. However, using AHA method and low pH method, the bacterial solution and cementing solution diffuse more evenly, increasing the nucleation sites of calcium carbonate. The growth of crystals is limited by space and growth substances, so their size is smaller than that of the control group. The reason why the crystal size of calcium carbonate in the low pH method is smaller than that in the AHA method is that in a lower pH environment, it can not only inhibit the activity of urease, but also delay the rate of carbonate generation, resulting in more and smaller calcium carbonate crystals that bond with sand particles to form a denser structure. When calcium carbonate crystals play a role in bonding and bridging between particles, the more calcium carbonate bonding points there are, the higher the strength. Therefore, the strength enhancement effect of these three reinforcement methods can be explained by the size and quantity of the crystals.

## 4 Mechanism of action

**Figure 13:**
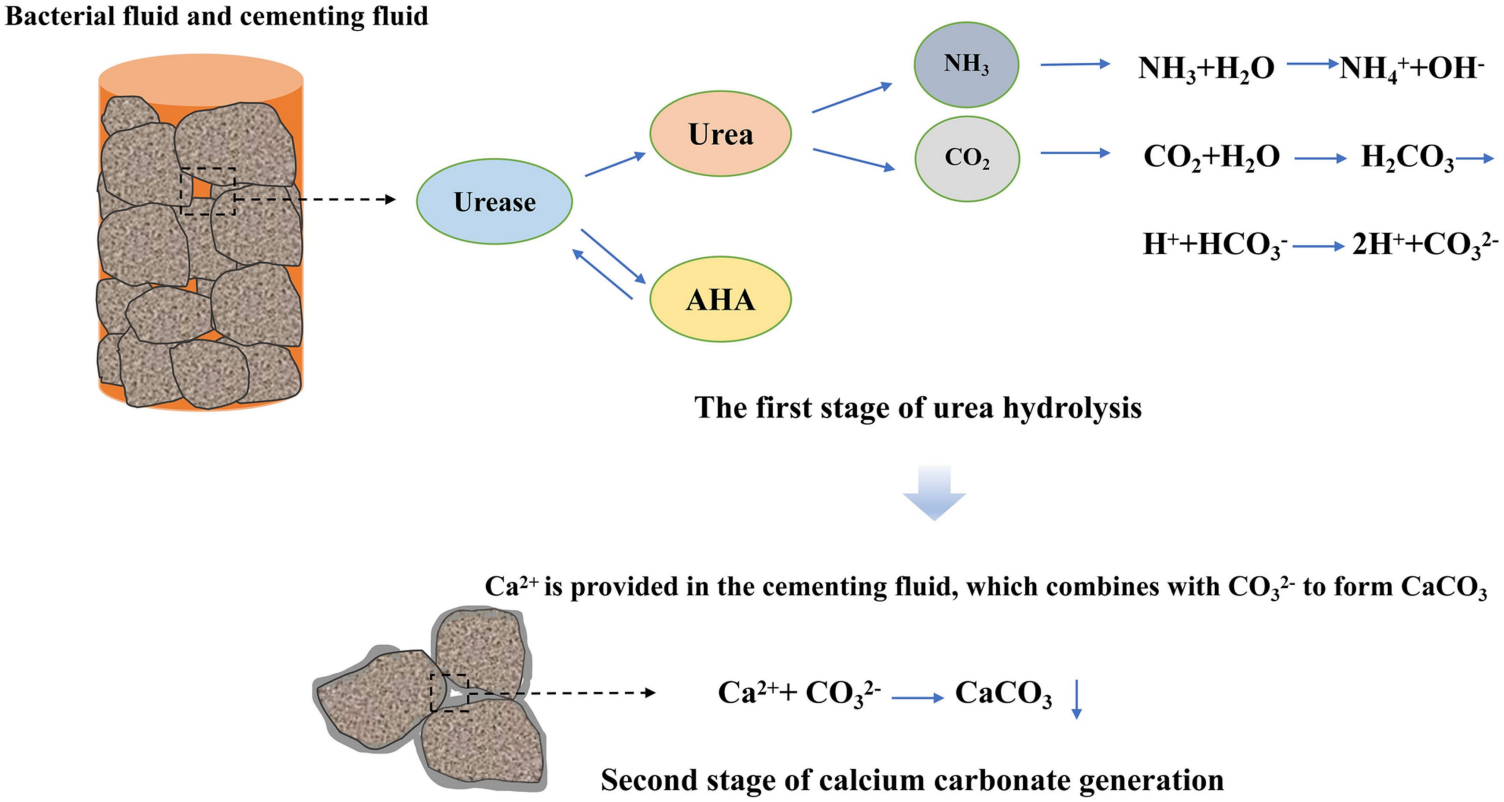
MICP reinforcement mechanism of urea hydrolysis.

MICP reinforcement of sandy soil can be divided into two stages: urea hydrolysis process and calcium carbonate deposition process, as shown in Figure 13. In the first stage, urea is decomposed into NH_3_ and CO_2_ under the action of urease; NH_3_ generates NH_4_^+^ and OH ^−^ under the action of water, and OH ^−^ gradually increases the pH value of the solution. CO_2_ is converted into H_2_CO_3_ and HCO_3_^−^ according to different pH values in the solution, and gradually converted into CO_3_^2−^ in alkaline environment. CO_3_^2−^ combines with Ca^2+^ in the cementing fluid to form calcium carbonate crystals, completing the second stage of calcium carbonate deposition process. Urease inhibitors act on the first stage of the binding process between urea and urease. Urease is a metalloenzyme, and the structural protein of intracellular urease produced by *Bacillus subtilis* contains three active centers, each containing two nickel ions. Urease is activated by nickel ions, allowing urea to enter and react with it. After AHA is added, it competes with urea to bind with the active site of urease and has exclusivity. Therefore, a portion of urease in the solution will react with urea, while another portion will react with AHA. AHA binds to the nickel ion in the active center of urease through its hydroxamic acid group, thereby preventing urea molecules from entering the active center and inhibiting urease activity. The inhibitory effect of AHA is reversible, and once the urease bound to AHA is disconnected, it can still react with urea. Therefore, urease inhibitors can delay the binding time between urease and urea, thereby inhibiting the hydrolysis rate of urea (Ciurli et al., 1996; Stemmler et al., 1995). The low pH method acts on the generation process of CO_3_^2−^ in the first stage, where NH_3_ decomposes into NH_4_^+^ in water, accompanied by the generation of OH ^-^. As a result, the pH value of the solution increases, and CO_2_ is converted into CO_3_^2−^ in an alkaline environment. At this point, the pH value of the solution is lowered, and in order to generate CO_3_^2−^, the pH value of the solution must be buffered first, thereby delaying the rate of CO_3_^2−^ generation and thus delaying the formation of calcium carbonate crystals. On the other hand, the activity of urease is also inhibited at lower pH. Under brief low pH conditions, the inhibition of urease activity is reversible, so the bacterial solution used in the experiment needs to be prepared and used immediately to avoid complete inactivation of urease.

## 5 Conclusion

This article comprehensively analyzes the experimental results of sand samples reinforced by AHA method and low pH MICP method in terms of water absorption rate, calcium carbonate generation rate, and unconfined compressive strength. Combined with the observation of failure morphology and microscopic point of view, the optimal reinforcement methods of AHA method and low pH method MICP are evaluated from a quantitative and qualitative point of view. The main conclusions are as follows:

1. Exploring the effects of AHA method and low pH method in MICP reinforcement, it was found that the two methods can improve the mechanical effect of microbial reinforcement of calcareous sand, and the improvement effect was the most significant when AHA content was 0.1% or pH value was reduced to 5.5.
2. From SEM analysis, the size of the calcium carbonate crystals generated by the low pH method was smaller than that of the AHA method and the blank control. At similar calcium carbonate contents, the smaller the crystal size, the more bonding points between calcium carbonate and soil, and the higher its strength.
3. By exploring the mechanisms of AHA method and low pH method MICP reinforcement of sandy soil, it was found that AHA method delays the formation rate of calcium carbonate by delaying the rate of urea hydrolysis catalyzed by urease, while low pH method acts on the process of carbonate ion binding with calcium ion after urea hydrolysis and inhibits urease activity to delay the formation rate of calcium carbonate. The two methods ultimately achieve the same goal, but their mechanisms of action are different.

The study can effectively alleviate the poor reinforcement effect caused by blocking the grouting hole in MICP solidified calcareous sand project, and provide important theoretical basis and technical support for the application of calcareous sand in soil improvement.

## Data Availability

The original contributions presented in the study are included in the article/supplementary material, further inquiries can be directed to the corresponding author.
